# PU.1 alleviates the inhibitory effects of cigarette smoke on endothelial progenitor cell function and lung-homing through Wnt/β-catenin and CXCL12/CXCR4 pathways

**DOI:** 10.18332/tid/174661

**Published:** 2024-01-25

**Authors:** Xue He, Yanan Cui, Tiao Li, Lijuan Luo, Zihang Zeng, Yiming Ma, Yan Chen

**Affiliations:** 1 Department of Thoracic Surgery, The Second Xiangya Hospital, Central South University, Changsha, China; 2 Department of Pulmonary and Critical Care Medicine, The Second Xiangya Hospital, Central South University, Changsha, China; 3 Research Unit of Respiratory Disease, Central South University, Changsha, China; 4 Diagnosis and Treatment Center of Respiratory Disease, Central South University, Changsha, China; 5 Department of Pulmonary and Critical Care Medicine, Center of Respiratory Medicine, China-Japan Friendship Hospital, Beijing, China

**Keywords:** PU.1, cigarette smoke extract, endothelial progenitor cell, Wnt/β-catenin pathway, CXCL12/CXCR4 axis

## Abstract

**INTRODUCTION:**

Endothelial progenitor cells (EPCs) dysfunction is involved in the pathogenesis of chronic obstructive pulmonary disease (COPD). The transcription factor PU.1 is essential for the maintenance of stem/progenitor cell homeostasis. However, the role of PU.1 in COPD and its effects on EPC function and lung-homing, remain unclear. This study aimed to explore the protective activity of PU.1 and the underlying mechanisms in a cigarette smoke extract (CSE)-induced emphysema mouse model.

**METHODS:**

C57BL/6 mice were treated with CSE to establish a murine emphysema model and injected with overexpressed PU.1 or negative control adeno-associated virus. Morphometry of lung slides, lung function, and apoptosis of lung tissues were evaluated. Immunofluorescence co-localization was used to analyze EPCs homing into the lung. Flow cytometry was performed to detect EPC count in lung tissues and bone marrow (BM). The angiogenic ability of BM-derived EPCs cultured *in vitro* was examined by tube formation assay. We determined the expression levels of PU.1, β-catenin, C-X-C motif ligand 12 (CXCL12), C-X-C motif receptor 4 (CXCR4), stem cell antigen-1 (Sca-1), and stemness genes.

**RESULTS:**

CSE exposure significantly reduced the expression of PU.1 in mouse lung tissues, BM, and BM-derived EPCs. PU.1 overexpression attenuated CSE-induced emphysematous changes, lung function decline, and apoptosis. In emphysematous mice, PU.1 overexpression markedly reversed the decreased proportion of EPCs in BM and promoted the lung-homing of EPCs. The impaired angiogenic ability of BM-derived EPCs induced by CSE could be restored by the overexpression of PU.1. In addition, PU.1 upregulation evidently reversed the decreased expression of β-catenin, CXCL12, CXCR4, Scal-1, and stemness genes in mouse lung tissues, BM, and BM-derived EPCs after CSE exposure.

**CONCLUSIONS:**

PU.1 alleviates the inhibitory effects of CSE on EPC function and lung-homing via activating the canonical Wnt/β-catenin pathway and CXCL12/CXCR4 axis. While further research is needed, our research may indicate a potential therapeutic target for COPD patients.

## INTRODUCTION

Chronic obstructive pulmonary disease (COPD) is a major cause of chronic morbidity and mortality worldwide^1^. Cigarette smoke exposure is the main trigger factor of emphysema^1^. Researchers have found that the imbalance between apoptosis and proliferation of septal endothelial cells due to a decreased expression of lung vascular endothelial growth factor (VEGF) and its receptor 2 (VEGFR2), termed also Flk-1, contributes to the destruction of alveolar structures and takes part in the pathogenesis of COPD^2^.

Endothelial progenitor cells (EPCs) are bone marrow (BM)-derived precursor cells which have the potential to differentiate into mature endothelial cells (ECs)^3^. Endothelial injury triggers the mobilization of EPCs from BM into the peripheral circulation. Circulating EPCs can home into sites of injury where they differentiate into ECs and participate in vascular repair^4^. Damaged cells of the alveolar wall can be replaced by migrating progenitor cells and depletion of these cells could lead to an impaired repair capacity^5^. Therefore, stem cell depletion might be a pathogenesis of emphysema^5^. The self-renewal and differentiation of stem/progenitor cells are regulated by master pluripotency (stemness) transcription factors sex determining region Y-box 2 (Sox2), octamer-binding transcription factor 4 (Oct4), and homeobox protein Nanog^6^.

EPCs are one of the main exhausted stem cells in COPD. Fadini et al.^7^ reported that EPCs (CD34^+^CD133^+^VEGFR2^+^) in peripheral blood detected by flow cytometry were closely related to the severity of COPD. Our previous study showed that in patients with COPD, circulating EPCs were reduced and dysfunctional^8^. In a murine emphysema model, we further found that the decreased expression of stem cell antigen-1 (Sca-1), a surface marker of stem/progenitor cells, was associated with cigarette smoke extract (CSE)-induced EPC dysfunction^9^. The mechanism for maintaining EPC homeostasis has not been fully understood. Research has revealed that in mesenchymal stem cells of COPD patients, the C-X-C motif ligand 12 (CXCL12)/C-X-C motif receptor 4 (CXCR4) chemokine axis was significantly inhibited^10^. The CXCL12/CXCR4 axis could be a key regulator of BM progenitor cell mobilization^11^. In addition, it was reported that the canonical Wnt/β-catenin pathway regulated EPC fate through metabolism^12^. Activated β-catenin induced the expression of stem cell self-renewal related genes^13^. However, the mechanism of regulating the specific role of CXCL12/CXCR4 interactions and Wnt signalling in normal EPC homeostasis is still less clear.

PU.1, a member of the erythroblast transformation specific (ETS) transcription factor family, is a DNA binding protein encoded by proto-oncogene Sfpi-1^14^. The expression of PU.1 is essential for the development of common progenitor of lymphoid and myeloid cell lineages in the hematopoietic system^15^. Kim et al.^16^ found that in the PU.1 knockout mouse model, the Sca-1^+^ hematopoietic stem cells (HSCs) and CD34^+^ progenitor cells were reduced in fetal liver, suggesting that PU.1 played an important role in the maintenance of HSC number. Moreover, transcription factor PU.1 may interact with Wnt/β-catenin signaling to regulate monocyte-macrophage differentiation^17^. However, the exact mechanisms underlying the effects of PU.1 on EPC function remain unclear and little information is focused on the role of PU.1 in COPD.

In the present study, we aimed to explore the effects of PU.1 on the function and lung-homing of EPCs and the expression of β-catenin, CXCL12, and CXCR4 in a CSE-induced murine emphysema model. Our results provided new insights into the role of PU.1 and EPC dysfunction in COPD, which might serve as a novel therapeutic target.

## METHODS

### Preparation of CSE

In accordance with the method proposed by Chen et al.^18^, 5 cigarettes (Marlboro, Philip Morris, USA) were burned and collected in a modified syringe-driven apparatus filled with 10 mL phosphate buffered saline (PBS) using a vacuum pump. Each cigarette contained 12 mg tar and 1 mg nicotine. The solution was then filtered through a microfilter with a pore size of 0.22 μM to obtain 100% CSE. The CSE solution was freshly prepared each time.

### Animal protocols

Forty male C57BL/6 mice (4–5 weeks) were purchased (Hunan Slyke Jingda Laboratory Animal Co., Ltd.) and randomly divided into four groups: the control group, CSE group, CSE + AAV-NC group, and CSE + AAV-PU.1 group, with ten mice in each group. The murine emphysema model was established as previously described with a slight improvement^19^. The control group and the other three groups were injected intraperitoneally with 0.3 mL PBS or 100% CSE per 20 g mouse, at days 30, 41, 52, and 63, respectively. The CSE + AAV-NC group and CSE + AAV-PU.1 group received a tail vein injection of 10^8^ ifu/mL (100 μL per mouse) negative control adeno-associated virus (AAV, Cyagen Biosciences) or PU.1 overexpression AAV (Cyagen Biosciences) at day 1, while mice in the control group and CSE group were injected with an equal volume of PBS via the tail vein at day 1. All mice were sacrificed at day 65. All animal experiments were conducted in accordance with the Animal Ethics Committee of the Second Xiangya Hospital of Central South University (Approval number: 2017218).

### Lung function

Lung function was measured using plethysmograph (Buxco Respiratory Products, USA) as previously described^20^. The respiratory frequency (F), tidal volume (TV), airway resistance (Raw), and dynamic lung compliance (Cdyn), were recorded. All operations were performed by the same technician at the Innovative Experimental Platform of Xiangya Medical College of Central South University in a blinded manner.

### Lung tissues preparation

The left lungs were inflated with 4% paraformaldehyde at a pressure of 25 cmH2O and then fixed in 4% paraformaldehyde for at least 24 hours followed by paraffin-embedded sections. The right lower lobes were collected for flow cytometry analyses and the other right lungs were stored in liquid nitrogen for subsequent measurements.

### BM collection and BM non-red blood cell isolation

As described by He et al.^21^, femurs and tibias were collected from each mouse. The joint end was cut off and the BM cavity was exposed. Then, 3 mL M199 (SH30253.01B, HyClone, USA) was used for each femur or tibia to flush the BM cavity. The flush fluid was pipetted up and down 30 times to break up the tissue into a cell suspension, which was immediately centrifuged for 10 min at 1500 rpm and 4^o^C. The supernatant was stored at -80^o^C for further analyses and the cell precipitation was resuspended with 3 mL M199. BM non-red blood cells were isolated from the resulting cell suspension by red blood cell lysis (CW0613, ComWin Biotech, China).

### Lung tissue morphometry and immunohistochemistry (IHC)

Paraffin-embedded lung tissues were cut into 4 μm thick sections and stained with hematoxylin and eosin (HE). We used the mean linear intercept (MLI) and the destructive index (DI) to assess the degree of emphysema^19^. IHC was conducted to explore the expression and localization of PU.1. Sections were incubated with anti-PU.1 (sc-390405, Santa Cruz Biotechnology, USA). The results were evaluated by the percentage of positive cells in each field.

### TUNEL analysis

The presence of apoptotic cells was assessed with terminal deoxynucleotidyl transferase-mediated dUTP nick-end labeling (TUNEL) assays using an apoptosis detection kit (G1502, Servicebio, China). The cells with red fluorescence were calculated as apoptotic cells and the apoptotic index (AI) was evaluated.

### Immunofluorescence staining

Sections were incubated with anti-CD34 (AF5149, Affinity Biosciences, USA), anti-CD133 (AF5120, Affinity Biosciences, USA), and anti-CD31 (GB13428, ServiceBio, China) at 4°C overnight. Then, sections were immunoblotted with fluorescence-conjugated secondary goat anti-rabbit antibodies. The nucleus was stained with 4’6-diamidino-2-phenylindole (DAPI). Finally, images were captured under a fluorescence microscope.

### Flow cytometry analysis

Suspended BM non-red blood cells and single-cell suspensions freshly obtained from collagenasedigested lung tissues were labeled with FITCconjugated anti-CD34 (560238, BD Biosciences, USA), PE-conjugated anti-CD133 (12-1331, eBioscience, USA), and APC-conjugated anti-Flk-1 (560070, BD Biosciences, USA) at 4^o^C for 30 min in the dark. Approximately, 5×10^5^ cells were analyzed by flow cytometry for each experiment using a FACSCalibur^TM^ flow cytometer (BD Biosciences, USA).

### Isolation, culture, and identification of EPCs

The mouse lymphocyte separation medium (10771, Sigma-Aldrich, USA) was used to isolate mononuclear cells from BM through density gradient centrifugation according to previously published method^22^. The isolated mononuclear cells were inoculated into culture flasks at a density of (2–4)×10^6^/mL and cultured with EGM-2MV containing 5% fetal bovine serum (CC-3202, Lonza, Switzerland) under an atmosphere of 95% humidity and 5% CO2 at 37^o^C. We conducted cell harvesting on day 7 of the culture. Double positive staining with ulex europaeus agglutinin-1 (UEA-1) and acetylated low-density lipoprotein (acLDL) was used for EPC identification. The harvested cells were firstly incubated with 7.5 μg/mL Dil-acLDL (L3484, Molecular Probes, USA) at 37^o^C for 4 h and later fixed with 4% paraformaldehyde for 10 min. After being washed, the cells were treated with 10.0 μg/mL FITC-UEA-1 (L9006, Sigma-Aldrich, USA) for 30 min. Finally, the cells were stained with DAPI before identification performed using an inverted fluorescence microscope.

### Tube formation assay

BM-derived EPC vascular formation ability was examined by performing Matrigel tube formation assay *in vitro*. After thawed on ice, Matrigel (354234, Corning, USA) was used to cover 48-well plates and re-solidified at 37^o^C for 30 min. Subsequently, BM-derived EPCs were dispensed into the plates at 5×10^4^ per well and incubated for 8 h. An inverted microscope (BX43, Olympus, Japan) was used to take photographs.

### Western blot analysis

Lung tissues were lysed in RIPA lysis buffer (Beyotime, China) to obtain total proteins. The protein concentration was determined by a BCA protein assay kit (Thermo Fisher Scientific, USA). Equal amounts of proteins were separated using sodium dodecyl sulfate polyacrylamide gel electrophoresis and transferred to polyvinylidene fluoride (PVDF) membranes. After being blocked for 1 h, PVDF membranes were incubated with specific primary antibodies against PU.1 (sc-390405, Santa Cruz Biotechnology, USA), Sca-1 (ab109211, Abcam, UK), β-catenin (8480, Cell Signaling Technology, USA), and β-tubulin (10068-1-AP, Proteintech, China) overnight at 4^o^C. Then, these membranes were incubated with anti-mouse or anti-rabbit IgG HRP-labeled secondary antibody (Proteintech, China) for one hour at room temperature. Labeled proteins were detected by the ECL plus Western blotting detection system (Bio-Rad, USA).

### Real-time quantitative polymerase chain reaction (RT-qPCR)

Total RNA was isolated from lung tissues, BM non-erythrocytic tissues, or BM-derived EPCs cultured *in vitro* using TRIzol reagent (Invitrogen, USA). RNA was then reversely transcribed into complementary DNA (cDNA) using a RevertAid First Strand cDNA Synthesis Kit (Thermo Fisher Scientific, USA). RT-qPCR was carried out using an All-in-One qPCR Mix kit (GeneCopoeia, China) according to the instructions of the manufacturer. Glyceraldehyde 3-phosphate dehydrogenase (GAPDH) was applied as an internal control. The sequences of all primers used in our study were:

PU.1-F: 5'-ATGTTACAGGCGTGCAAAATGG-3'; PU.1-R: 5'-TGATCGCTATGGCTTTCTCCA-3'; β-catenin-F: 5'-ATGGAGCCGGACAGAAAAGC-3'; β-catenin-R: 5'-CTTGCCACTCAGGGAAGGA-3'; CXCR4-F: 5'-GAAGTGGGGTCTGGAGACTAT-3'; CXCR4-R: 5'-TTGCCGACTATGCCAGTCAAG-3'; Sox2-F: 5'-GCGGAGTGGAAACTTTTGTCC-3'; Sox2-R: 5'-CGGGAAGCGTGTACTTATCCTT-3'; Oct4-F: 5'-CGGAAGAGAAAGCGAACTAGC-3'; Oct4-R: 5'-ATTGGCGATGTGAGTGATCTG-3'; Nanog-F: 5'-TCTTCCTGGTCCCCACAGTTT-3'; Nanog-R: 5'-GCAAGAATAGTTCTCGGGATGAA-3'; GAPDH-F: 5'-AGGTCGGTGTGAACGGATTTG-3'; and GAPDH-R: 5'-TGTAGACCATGTAGTTGAGGTCA-3'.

### Enzyme-linked immunosorbent assay (ELISA)

CXCL12 levels in the supernatant of BM flush fluid were measured using an ELISA kit (DY460, R&D Systems, USA) as described by the manufacturer’s instructions.

### Statistical analysis

Continuous data are expressed as mean ± standard deviation (SD). Statistical comparisons were performed using the one-way ANOVA combined with the Tukey’s post hoc test. GraphPad Prism (GraphPad Prism 8.0, USA) was used to analyze the data. A two-sided p<0.05 was considered statistically significant.

## RESULTS

### Expression of PU.1 in mice

**Figure 1:**
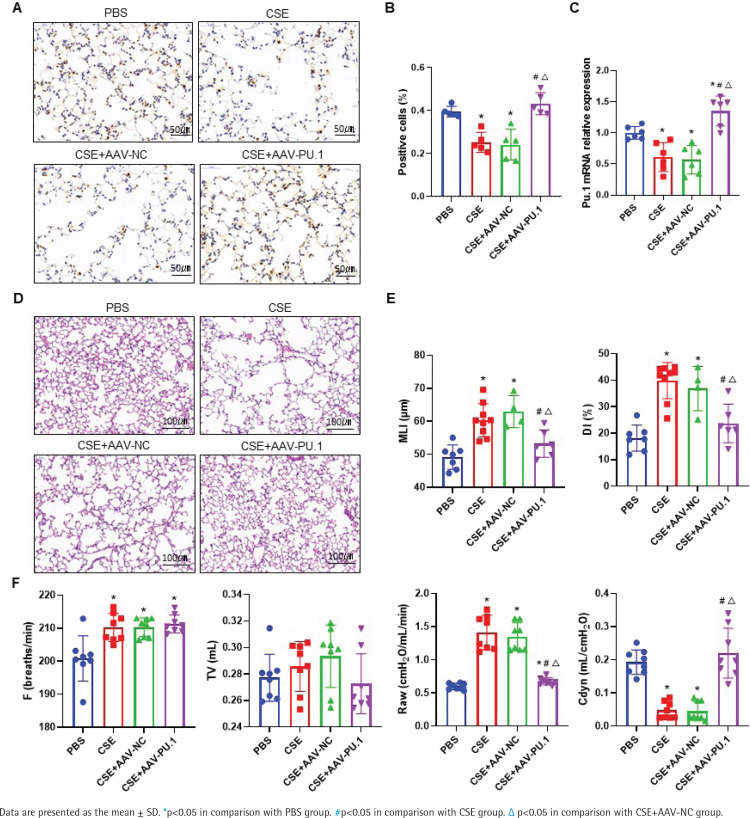
PU.1 alleviates CSE-induced lung histological and functional changes in mice. A) IHC staining of PU.1 in mouse lung tissues. Scale bars represent 50 μm. Dark brown nuclear cells were positive cells; B) Percentages of positive cells in IHC staining; C) Levels of PU.1 mRNA in mouse lung tissues; D) HE staining of lung slides. Scale bars represent 100 μm; E) Morphometric measurements of MLI (μm) and DI (%); F) Lung function measurements of respiratory frequency (F, breaths/min), tidal volume (TV, mL), airway resistance (Raw, cmH2O/mL/min), and dynamic lung compliance (Cdyn, mL/cmH2O)

Transcription factor Pu.1 regulates gene transcription and expression in the nucleus. The dark brown nuclear positive cells using IHC represented the expression of PU.1 (Figure 1A). Compared with the PBS group, the proportion of positive cells in the CSE group and CSE + AAV-NC group was markedly decreased, suggesting that the PU.1 protein level was significantly reduced in lung tissues of mouse with emphysema (Figure 1B). After intervention with PU.1 overexpression AAV, the expression levels of PU.1 protein (Figure 1B) and mRNA (Figure 1C) were both significantly increased compared with the CSE + AAV-NC group, indicating a successful overexpression of PU.1 in mice.

### PU.1 alleviates CSE-induced lung histological and functional changes in mice

HE staining was performed to detect histological changes in mouse lung tissues (Figure 1D). In the CSE group, the alveolar space was enlarged and the lung parenchyma was destroyed. PU.1 overexpression significantly attenuated the degree of emphysema. The MLI and DI values were significantly increased in the CSE group compared with the PBS group, but these values were reduced in the CSE + AAV-PU.1 group compared with the CSE + AAV-NC group (Figure 1E). The F, TV, RAW, and Cdyn, were measured to reflect the resistance of small airway and elasticity (Figure 1F). Compared with the PBS group, the RAW in the CSE group and CSE + AAV-NC group was significantly increased and the Cdyn was significantly decreased, which indicated that there was obstructed airflow in our mice model. However, PU.1 overexpression significantly improved the lung function damage induced by CSE.

### PU.1 attenuates CSE-induced apoptosis in mice

TUNEL analyses were performed to clarify whether the reduced PU.1 was associated with cell apoptosis in lung tissues of CSE-exposed mice (Supplementary file Figure S1A). Significantly more TUNEL-positive cells in alveolar septum were observed in the CSE group and CSE + AAV-NC group compared with the PBS or CSE + AAV-PU.1 group, suggesting that PU.1 could attenuate CSE-induced cell apoptosis (Supplementary file Figure S1B).

### PU.1 ameliorates the inhibitory effect of CSE on lung-homing of EPCs in mice

**Figure 2:**
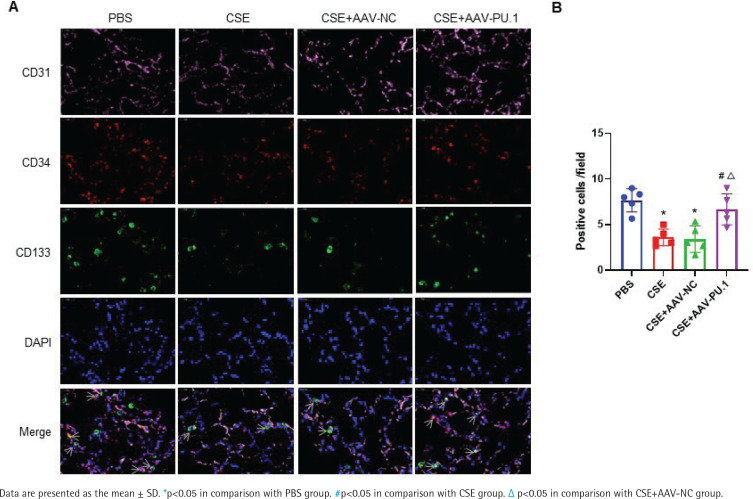
Immunofluorescence co-localization in mouse lung tissues. A) Immunofluorescence staining of CD31 (pink), CD34 (red), and CD133 (green) and DAPI staining of nuclei (blue) at ×900 magnifications. → the EPCs co-expressing CD31, CD34, and CD133; B) Statistical analyses of the number of positive cells (EPCs) in each field

In lung tissues, cells showing the co-localization of CD34, CD133, and CD31 were identified as EPCs homing into the lung using immunofluorescence staining (Figure 2A). Recruited EPCs were mainly distributed in the pulmonary microvascular area in alveolar septum. The number of EPCs homing into the lung was significantly lower in the CSE group and CSE + AAV-NC group than in the PBS group (Figure 2B). However, PU.1 overexpression markedly promoted EPC recruitment into the lung in the CSE-exposed mouse model (Figure 2B).

**Figure 3:**
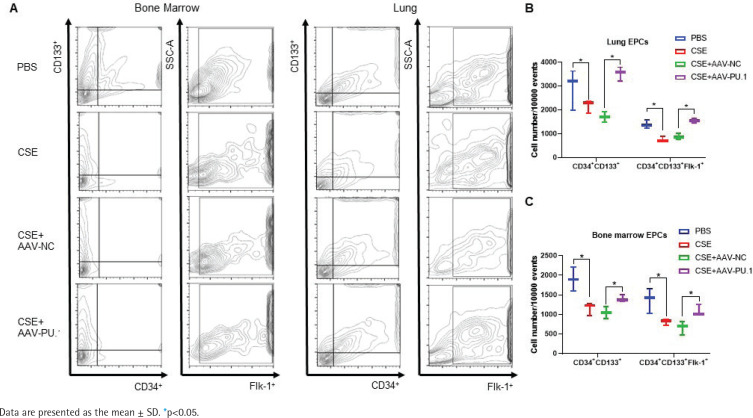
Flow cytometry in mouse bone marrows and lung tissues. A) Flow cytometry analyses to identify EPCs in bone marrow non-erythrocytic tissue monocyte suspensions and lung tissue single-cell suspensions; B) Statistical analyses of proportions of CD34^+^CD133^+^ cells and proportions of CD34^+^CD133^+^Flk-1^+^ cells in lung tissues; C) Statistical analyses of proportions of CD34^+^CD133^+^ cells and proportions of CD34^+^CD133^+^Flk-1^+^ cells in bone marrows

We also detected the proportions of CD34^+^CD133^+^ cells and CD34^+^CD133^+^Flk-1^+^ EPCs in lung tissues and BM by flow cytometry (Figure 3A). CSE exposure caused a significant inhibition of CD34^+^CD133^+^ cell numbers and CD34^+^CD133^+^Flk-1^+^ EPC numbers in both lung tissues and BM (Figures 3B and 3C). Upregulation of PU.1 level in CSE-exposed mouse effectively stimulated EPC recruitment into the lung (Figure 3B). In BM, PU.1 overexpression also significantly reversed the decreased proportion of EPCs induced by CSE, which might contribute to the increase in lung-homing of EPCs (Figure 3C).

### Identification of mouse BM-derived EPCs

**Figure 4:**
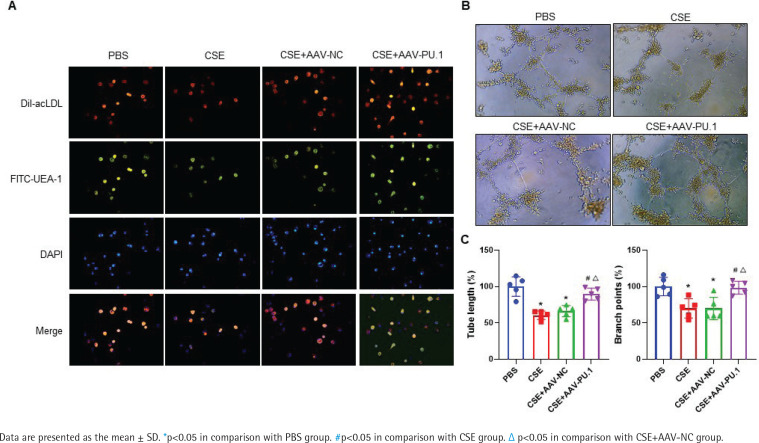
The identification of mouse bone marrow-derived EPCs and tube formation assay. A) The identification of bone marrow-derived EPCs of mice in each group by double positive staining with Dil-acLDL and FITC-UEA-1 at ×400 magnifications. The inverted fluorescence microscope demonstrated that the cells displayed red cytoplasm while taking up Dil-acLDL on day 7 of the culture, green cytomembrane when binding FITC-UEA-1, and blue when staining with DAPI in nuclear localization. The merged figures indicate cells with double positive staining of Dil-acLDL and FITC-UEA-1; B) Bone marrow-derived EPC tube formation in Matrigel on day 8 of the culture at ×400 magnifications; C) Relative tube length and number of branch points

The LSCM test showed that cells displayed red when taking up Dil-acLDL and green when combining with FITC-UEA-1 (Figure 4A). In all the four groups, large proportions of cells cultured *in vitro* were amphophilic cells.

### PU.1 alleviates the inhibitory effect of CSE on tube formation of mouse BM-derived EPCs

We measured the vascular formation ability of BM-derived EPCs cultured *in vitro* (Figure 4B). The data showed that the relative tube length and number of branch points of the cells were evidently reduced in the CSE and CSE + AAV-NC groups compared with the PBS group, suggesting that the angiogenic ability of EPCs in emphysematous mice was impaired (Figure 4C). However, such an inhibitory effect of CSE was reversed by overexpression of PU.1 (Figure 4C).

### PU.1 reverses the effects of CSE on the Wnt/β-catenin pathway, CXCL12/CXCR4 axis, Scal-1 and stemness genes expression

**Figure 5:**
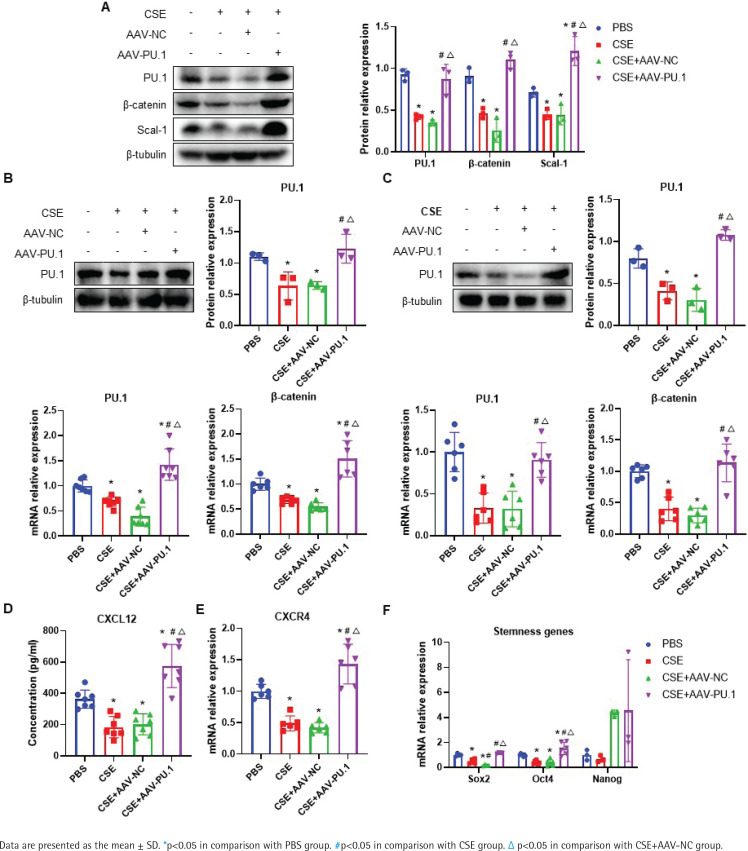
Effect of PU.1 on β-catenin level, CXCL12/CXCR4 axis, Scal-1 and stemness genes expression. A) Western blot analyses of PU.1, β-catenin, and Scal-1 protein levels in lung tissues; B) Expression levels of PU.1 and β-catenin in bone marrow non-erythrocytic tissues; C) Expression levels of PU.1 and β-catenin in bone marrow-derived EPCs; D) CXCL12 levels in the bone marrow microenvironment using ELISA assays; E) Levels of CXCR4 mRNA in bone marrow-derived EPCs; F) Levels of Sox2, Oct4, and Nanog mRNA in bone marrow-derived EPCs

In emphysematous mice, PU.1 protein and mRNA expression were inhibited in lung tissues, BM, and BM-derived EPCs cultured *in vitro* (Figures 5 A, B and C). The protein levels of β-catenin in the CSE and CSE + AAV-NC groups were significantly decreased in comparison with the PBS group in mouse lung tissues (Figure 5A). Meanwhile, intraperitoneal injection of CSE significantly decreased the mRNA expression level of β-catenin in both BM (Figure 5B) and BM-derived EPCs (Figure 5C). After upregulation of PU.1 level in mice, the inhibitory effect of CSE on the expression of β-catenin was markedly reversed.

The level of CXCL12 was detected in the supernatant of BM. As shown in Figure 5D, CSE could significantly reduce CXCL12 level, which was dramatically reversed after PU.1 upregulation. We then detected the expression of its specific receptor CXCR4 in BM-derived EPCs. Figure 5E shows that PU.1 overexpression evidently reversed the decreased level of CXCR4 mRNA induced by CSE.

Stem cell surface antigen and stemness transcription factor expression were measured to investigate the effect of PU.1 on CSE-induced EPC dysfunction. In lung tissues, PU.1 overexpression significantly reversed the inhibitory effect of CSE on Scal-1 protein level (Figure 5A). In BM-derived EPCs cultured *in vitro*, CSE exposure resulted in decreased mRNA levels of stemness-related genes Sox2 and Oct4 (Figure 5F). In addition, overexpression of PU.1 could obviously reverse the effect of CSE on Sox2 and Oct4 expression. Stemness gene Nanog expression showed no significant difference between each group.

## DISCUSSION

We revealed that lung-homing of EPCs and PU.1 expression were significantly inhibited in CSE-induced murine emphysema model. Moreover, we first found that PU.1 overexpression could attenuate CSE-induced emphysema and apoptosis and alleviate the inhibitory effects of CSE on EPC mobilization, lung-homing, and function in mice. In addition, we propose that PU.1 reversed the harmful effects of CSE by activating the canonical Wnt/β-catenin pathway and CXCL12/CXCR4 axis.

EPCs express a variety of markers with different intensity, which are typical for the endothelial lineage^23^. Different markers may be selected in different studies to identify EPCs. CD34 is widely used as a specific marker of hematopoietic cells. Asahara et al.^3^ first isolated CD34^+^ cells that could differentiate into ECs. CD133, an early stem cell marker, is expressed in hematopoietic stem and progenitor cells from human BM, fetal liver, and peripheral blood^24^. VEGFR2 is the principal receptor transmitting VEGF signals and also expressed in endothelial cell precursors and developing endothelial cells^25^. CD34^+^CD133^+^VEGFR2^+^ phenotype remains the most commonly recognized profile for EPCs. Furthermore, CD31, CD45, CD117, and Tie2 have also been identified as markers of EPCs^26^. In our study, two methods using different combination of EPC markers were implemented to quantify EPCs. CD34^+^CD133^+^Flk-1^+^ EPCs were enumerated by flow cytometry and CD34^+^CD133^+^CD31^+^ EPCs were counted using immunofluorescence staining. In addition, previous studies reported that intratracheal transplantation of EPCs attenuated emphysema development by decelerating apoptosis^27^. We further found that the distribution of EPCs homing into the lung was consistent with the distribution of apoptotic cells, indicating that EPCs might directly act on the pulmonary microvascular area where cell apoptosis occurred and could accelerate proliferation and repair of epithelial cells in alveolar septum.

PU.1, a widely expressed transcription factor, has been studied as a critical regulator of genes involved in hematopoietic cell growth and immune response^14^. In PU.1 knockout mice, embryos died at about embryonic day 18.5 and showed a complete absence of B cells, mature T cells, and macrophages^16^. Overexpression of PU.1 could upregulate the expression of Sca-1 and affect HSC differentiation^28^. However, detailed molecular mechanisms of PU.1 in regulating EPC dysfunction and the role of PU.1 in COPD remain to be discovered.

In our experiments, stem cell surface antigen Scal-1 was down-regulated in lung tissues of emphysematous mice. BM-derived EPCs of emphysematous mice showed a decreased expression of stemness-related genes and a suppressed ability of angiogenesis. Thus, in a CSE-induced murine emphysema model, we found not only reduced EPC mobilization and lung-homing, but also impaired EPC function. Interestingly, the transcription factor PU.1 could reverse these harmful effects caused by CSE. Our previous work also showed that EPCs treated with CSE *in vitro* displayed decreased capacities of proliferation, adhesion, and secretion^29^. Moreover, extensive data have established that circulating EPCs are decreased in COPD subjects^30^, but the enumeration of EPCs homing into the lungs of COPD patients has not been studied. In this study, we explored the EPCs homing into the lung and investigated the protective effect of PU.1 for the first time in CSE-induced emphysematous mice. This may provide a novel therapeutic option for COPD patients.

The canonical Wnt/β-catenin pathway, also known as β-catenin-dependent signaling pathway, regulates gene transcription through the major signal transducer β-catenin^31^. Studies have revealed that Wnt/β-catenin signaling is inactivated in COPD patients and emphysematous mice, and its reactivation attenuates the pathological changes of emphysema models^32^. The Wnt/β-catenin pathway plays an essential role in the maintenance of HSC homeostasis *in vitro* and in vivo^13^. Shao et al.^12^ further showed that canonical Wnt signaling could modulate the expression of stemness genes (Oct4, Nanog, Sox2, and Txb3) in EPCs. However, few researchers have paid attention to the relationship between PU.1 and Wnt signalling. We found that PU.1 upregulation could activate the Wnt/β-catenin pathway and protect against the EPC dysfunction caused by CSE. Based on the above evidence, the activation of canonical Wnt signaling may be the underlying mechanism of PU.1 resisting CSE-induced EPC dysfunction.

CXCL12 is the first chemoattractant reported for human CD34^+^ progenitor cells^33^. By local injection of CXCL12 into athymic ischemic hindlimb muscle of nude mice combined with human EPC transplantation, Yamaguchi et al.^34^ proposed that locally delivered CXCL12 enhanced angiogenesis by augmenting EPC recruitment in ischemic tissues. In addition, Liu et al.^35^ demonstrated that CXCR4, the receptor of CXCL12, was substantially down-regulated in EPCs isolated from COPD patients. Interestingly, a recent study found that CXCL12 affected the transformation of BM stromal cells in a manner mediated by canonical Wnt signaling^36^. In our study, PU.1 overexpression activated both the CXCL12/CXCR4 axis and the canonical Wnt/β-catenin pathway. Therefore, the CXCL12/CXCR4 axis may be a critical pathway for the protective effects of PU.1 on EPCs.

Although it has been reported that transplantation of EPCs poses as a viable route of therapy for COPD^30^, more studies are needed to confirm potential mechanisms by which EPCs may exert a protective role in COPD pathogenesis. Furthermore, a more thorough understanding is required with respect to the molecular mechanisms of decreased PU.1 production in COPD patients and how this affects EPC count and function. These efforts may provide data for the engineering of cellular therapeutic agents in the field of regenerative medicine.

## CONCLUSIONS

Through animal experiments and *in vitro* cell experiments, the present study indicates that PU.1 alleviates the inhibitory effects of CSE on EPC function and lung-homing via activating the canonical Wnt/β-catenin pathway and CXCL12/CXCR4 axis. This study elucidates a novel molecular mechanism of EPC regulation and may indicate a novel therapeutic option for COPD patients.

## Data Availability

The data supporting this research are available from the authors on reasonable request.
