# Supplementary material for: PU.1 alleviates the inhibitory effects of cigarette smoke on endothelial progenitor cell function and lung-homing through Wnt/β-catenin and CXCL12/CXCR4 pathways
Source: Tob Induc Dis. 2024 Jan 25;22:10.18332/tid/174661. doi: 10.18332/tid/174661 (PMC10809061; doi:10.18332/tid/174661)
Supplement: Supplementary file 1 [file TID-22-27-s1.pdf]

Supplementary Material

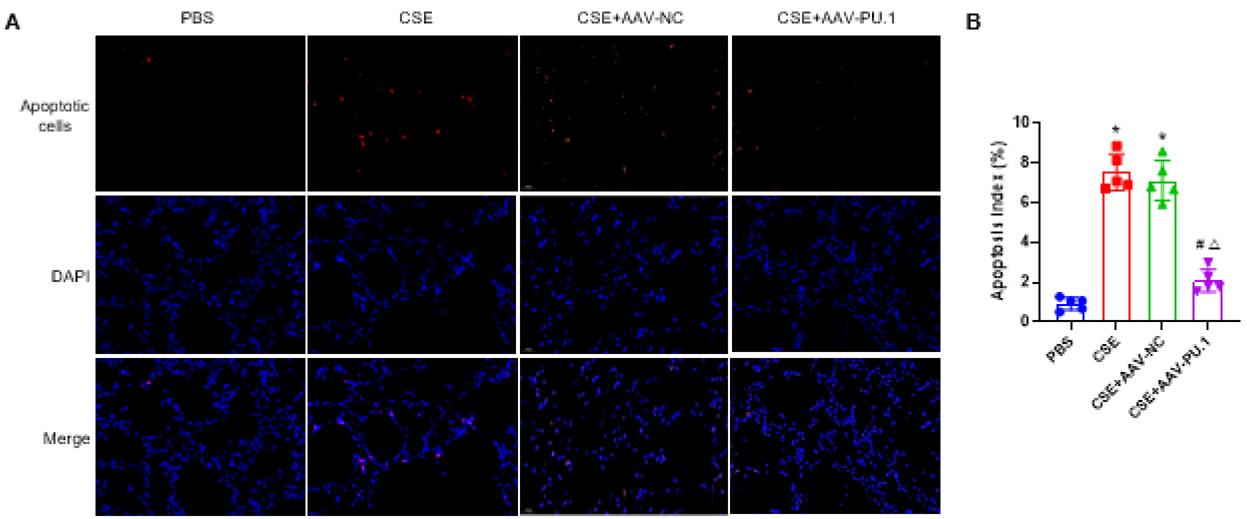

Figure S1 TUNEL staining of mouse lungs. (A) TUNEL staining of apoptotic cells (red) and DAPI staining of nuclei (blue) at ×400 magnifications. (B) Statistical analyses of apoptosis indexes. Data are presented as the mean ± SD. *P<0.05 in comparison with PBS group. #P<0.05 in comparison with CSE group. △P<0.05 in comparison with CSE+AAV-NC group.

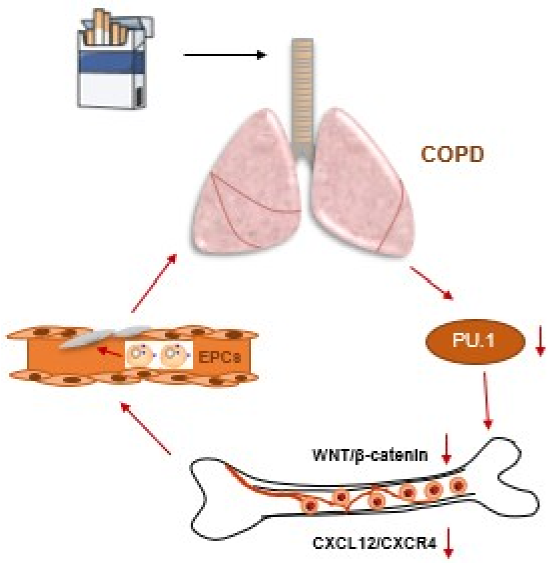

Figure S2 PU.1 regulates cigarette smoke induced dysfunction and reduced lung homing of EPCs via the classical Wnt/β-catenin pathway and CXCL12/CXCR4 axis.

Figure S3

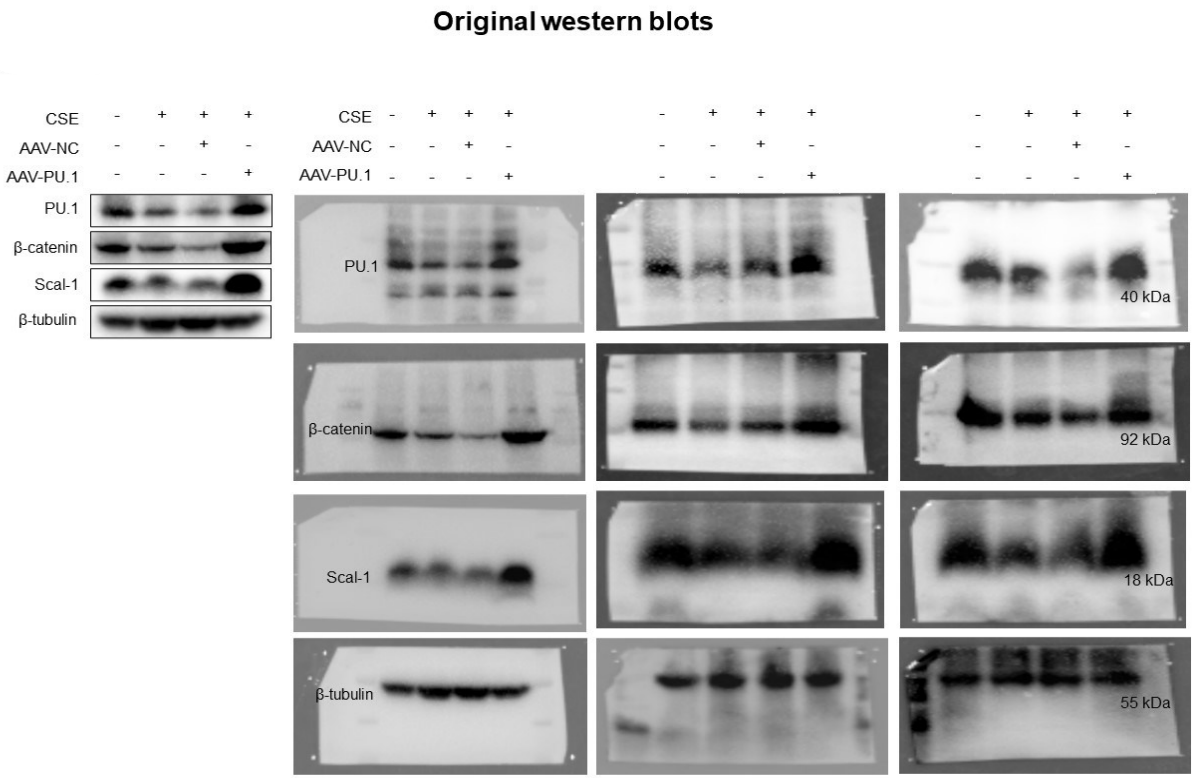
Original western blots
CSE
AAV-NC
AAV-PU.1
PU.1
β-catenin
Scal-1
β-tubulin
40 kDa
92 kDa
18 kDa
55 kDa

Figure S4

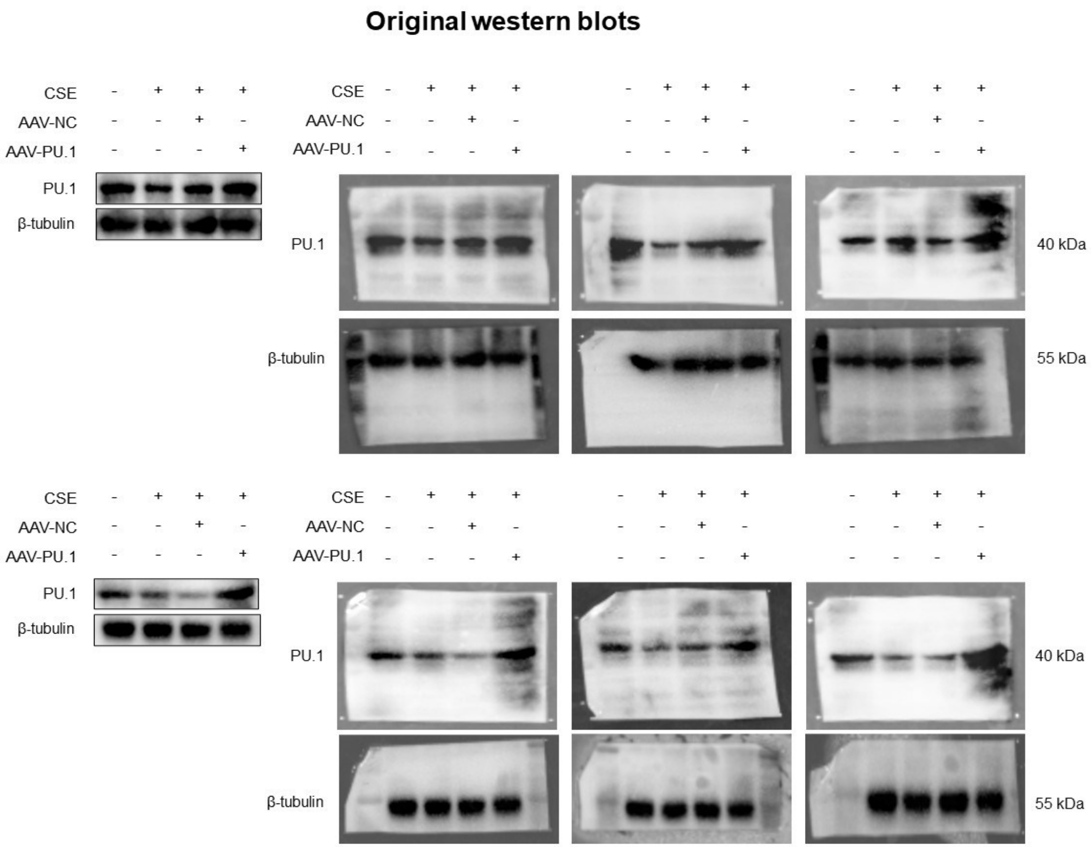

© 2024 He X. et al.
